# Standardization of imaging methods for machine learning in neuro-oncology

**DOI:** 10.1093/noajnl/vdaa054

**Published:** 2021-01-23

**Authors:** Xiao Tian Li, Raymond Y Huang

**Affiliations:** Department of Radiology, Brigham and Women’s Hospital, Boston, Massachusetts, USA

**Keywords:** machine learning, neuro-oncology, radiomics, reproducibility, standardization

## Abstract

Radiomics is a novel technique in which quantitative phenotypes or features are extracted from medical images. Machine learning enables analysis of large quantities of medical imaging data generated by radiomic feature extraction. A growing number of studies based on these methods have developed tools for neuro-oncology applications. Despite the initial promises, many of these imaging tools remain far from clinical implementation. One major limitation hindering the use of these models is their lack of reproducibility when applied across different institutions and clinical settings. In this article, we discuss the importance of standardization of methodology and reporting in our effort to improve reproducibility. Ongoing efforts of standardization for neuro-oncological imaging are reviewed. Challenges related to standardization and potential disadvantages in over-standardization are also described. Ultimately, greater multi-institutional collaborative effort is needed to provide and implement standards for data acquisition and analysis methods to facilitate research results to be interoperable and reliable for integration into different practice environments.

The field of artificial intelligence (AI), particularly machine learning, enables analysis of medical imaging data and helps augment current diagnostic imaging practice in the era of increasing clinical demand.^1^ AI algorithms are currently being researched to solve a number of clinical problems.^2^ In neuro-oncology, machine learning tools have been utilized for the development of radiomics models for tumor diagnosis and therapeutic response using noninvasive imaging techniques such as MRI and positron emission tomography (PET). Furthermore, radiomics can sometimes be categorized into predefined feature-based techniques versus deep learning-based techniques that do not require predefinition or the intermediate feature extraction step.^3,4^ Despite the promises of this developing field, most radiomic tools remain far from clinical implementation due to a number of challenges such as technical complexity, poor study design, overfitting of data, and lack of standards for validating results.^5,6^ The purpose of this article is to review current efforts of standardization for neuroimaging, more specifically to discuss the advantages of standardized methodology and reporting within radiomic and neuro-oncology research. Challenges related to standardization and potential disadvantages in over-standardization are also discussed.

## Need for Imaging Standardization

Advances in radiomic and machine learning methods have led to rapidly increasing efforts to generate automated tools and to help solve clinical problems in medicine. The field of radiomics is a swiftly developing novel technique in which image-based phenotypes of lesions including shape, size, and texture are extracted from medical images.^7^ A growing number of studies based on radiomic and machine learning methods have aimed at several neuro-oncology applications, including preoperative classification of central nervous system neoplasms including their histology, grade, and molecular signatures, as well as evaluating treatment response for better determination of treatment efficacy and prediction of subsequent clinical outcome. For example, machine learning models based on radiomic features of preoperative MRI have been developed to predict IDH1/2 mutations in both high- and low-grade gliomas.^8,9^ Classification models have also been developed to distinguish “pseudoprogression” from true tumor progression in glioblastomas receiving chemoradiation treatment.^10^ Other studies have shown that machine learning classifier can predict the tumor type of brain metastases.^11,12^ Studies in radiomics with additional modalities such as PET have gained increasing attention and shown to differentiate brain tumor or metastasis versus radiation injury,^13,14^ lymphoma versus glioblastoma,^15^ or predict underlying mutational status.^16^ Advanced MRI techniques have also incorporated radiomics such as MR perfusion for the identification of pseudoprogression in glioblastoma.^17^

Many researchers are increasingly automating the pipelines to generate radiomic models in order for easier integration into clinical use. A majority of these pipelines consist of numerous steps such as imaging acquisition and postprocessing. When examining the methods by which most of these models are constructed, one can readily discover that there is wide variability in every step leading to the final model. Variations in methodology among studies using radiomic approach often include patient demographics, patient selection criteria, patient cohort design, molecular or pathology data generation, imaging acquisition, image preprocessing, lesion segmentation, radiomic feature extraction, and machine learning computation. Even a small change in one or more of these key steps can result in significant differences in model accuracies and reproducibility. Due to these variations in the methodology, it is often difficult to show that one set of results are better or worse compared to the others by only directly comparing accuracies. Similarly, it is also not clear whether the results from one study can be applied to data obtained at another institution. Finally, a significant part of the challenge in determining the reproducibility of machine learning models is the frequent lack of sufficient methodology details in the published studies.

Due to the rapid development and application of radiomics methods, there is a lack of a standardized approach to analysis and reporting. To date, only a small number of studies that developed radiomic models for cancer care investigated their repeatability and reproducibility and provided details related to imaging processing and feature extraction.^18^ While researchers often employ cross-validation or include independent validation datasets to show reproducibility of trained radiomic models,^19^ the generalizability of these models can still be limited if they are applied to datasets that are sufficiently different from those that are used in training and validation. Thus, the development and implementation of standard imaging data acquisition methods can reduce variations that limit generalizability.

## Sources of Variability in Radiomic Methodology

Radiomics is a complex multistep process that requires a close examination of each of these steps in order to investigate sources of variability and to improve the reproducibility of the end results.

### Patient Cohort and Study Design

Within a different patient population, the type and prevalence of certain imaging findings and underlying pathology may be variable. One study with a homogenous patient cohort may get completely different results compared to another patient cohort with a more diverse underlying population. The inclusion and exclusion criteria for patient selection should therefore be clearly defined and potential biases related to these selection criteria should be discussed. In neuro-oncology, recent updates to the World Health Organization CNS tumor classification include molecular data in defining tumor subtypes.^20^ It is important to include details related to these molecular markers for the study population so that the resultant radiomic/AI models can be correctly applied clinically according to the updated criteria. Due to these changes in disease definitions, one should also be cautious in comparing radiomic models that were constructed using different disease criteria.

### Imaging Acquisition

Imaging acquisition methods including scanner types and sequence parameters can affect radiomic feature calculations. Studies show that computed tomography (CT) features may be nonreproducible and redundant by comparing 5 different scanners with the same CT parameters.^21^ Unlike CT where pixel intensities are directly related to tissue attenuation, the intensity values derived from MRI typically do not have physical meaning and widely vary depending on acquisition parameters and this results in significant variations among radiomic features derived from MRI.^22^ Based on MRI phantom analyses of commonly used MRI sequences, a study showed that FLAIR sequence is the most robust, although only 15 out of 45 radiomic features showed excellent robustness and reproducibility across all sequences.^23^ Evaluation of imaging protocol variability using a PET phantom demonstrates at least good reliability when parameters are in clinical range though there should be caution combining patients from different scanners into the same radiomics dataset.^24^ Another study using a PET phantom concluded that the repeatability of PET radiomic features depends on a number of factors and recommends high-level image acquisition and preprocessing standardization.^25^

### Imaging Preprocessing

Several imaging preprocessing steps are often performed prior to radiomic feature extraction in order to minimize intensity-related variations. These include noise filtering, inhomogeneity correction, and intensity normalization. When preprocessing steps are applied to MRI dataset, the robustness of radiomic features has improved based on phantom analyses^26^ as well as data from patients with glioblastoma.^27^

Intensity standardization or normalization is a preprocessing technique that changes the values of pixel intensity using a predefined algorithm in order to reduce variations from data acquisition. There are several normalization methods that can affect the reproducibility of radiomic features.^28^ Histogram matching is a commonly used normalization method and has been shown to contribute more than other preprocessing steps in reducing feature variability of conventional MRI sequences.^29^

### Tumor Segmentation

Segmentation of lesions is an important step to focus feature extraction on imaging pixels that are most relevant to the pathological process of interest and excluding other tissue types such as normal brain or treatment-related changes. Different lesion segmentation methods can introduce variability in subsequent feature calculations. For manual segmentation, it has been shown that interobserver variability in segmentation does impact radiomic analysis.^30^ Newer techniques of segmentation are developed to shorten the processing time. Semiautomatic techniques such as using software like 3D Slicer and TumorPrism3D have shown to reduce variability between observers.^31,32^ One study claims that interactive methods decrease variability compared to semiautomatic methods in segmenting glioblastomas.^33^ The Multimodal Brain Tumor Image Segmentation Benchmark (BRATS) introduced a large public dataset for the purpose of evaluating brain tumor segmentation algorithms with annual challenges.^34^ This benchmarking resource allowed the testing of a number of algorithms including fully automated ones. The development of a fully automated segmentation technique would tremendously benefit clinical care and treatment planning. Initial results showed that different algorithms, including fully automated ones, showed promise for different subregions of segmentation, but no single algorithm was top tier in all of the subregions^34^ in comparison to expert manually annotated results. Fully automated techniques are technically challenging partly due to variations in tumor structures and the displacement of normal structures due to mass effect.^34^ Since 2015, deep learning-based models, specifically convolutional neural networks (CNN), have been the top-performing techniques based on BRATS benchmarking.^35^ Many different deep learning techniques have been used including ensembles and a variety of neural networks.^36^ Recently, CNN models trained using sequential MRI data provide automated segmentation of patients with glioblastoma in clinical trials and provide objective response assessment comparable to expert evaluation.^37,38^

### Radiomic Feature Extraction and Selection

Given a data type, some radiomic features show greater robustness compared to other features. Therefore, it is often advantageous to use the features with greater robustness to improve model reproducibility. In the textural analysis of MRI from patients with a brain tumor, many features derived from a standard radiomic library lack robustness.^39^ Many radiomic features are shown to not be robust in the setting of different matrix size and dynamic range configurations.^40^ Without standardization of matrix size and dynamic range configurations, it would be challenging to obtain reliable and comparative results across different centers.^39^ Initial evaluation of feature variability in FDG-PET showed that many of the radiomic features do have high retest and interobserver stability with increased robustness seen in features more stable in repeated PET imaging.^41^ Gray-level co-occurrence matrix and shape features have been shown to be least sensitive to PET imaging system variations.^42^

Techniques have been developed to increase the robustness of radiomic features. One commonly used method is to introduce perturbations to images to help select robust features that are not susceptible to these perturbations.^43^ Automated machine learning systems have also been developed to scan for novel features and optimize parameters.^44^ There are methods to assess the stability and discriminatory capacity of radiomic features on apparent diffusion coefficient images.^45^

### Machine Learning Algorithms

As different machine learning approaches are used for neuroimaging, the types of models used will impact the variability. While it is possible that with sufficient optimization of machine learning hyperparameters, the performance of different algorithms can be equivalent. In practice, however, the fine-tuning process of these parameters can vary due to differences in expertise. The difference in the choice of classification method can be a dominant source of performance variation.^46^ The recent development in automatic machine learning algorithms such as Tree-based Pipeline Optimization Tool can select the best performing machine learning algorithms or hybrid algorithms.^47^ This automatic machine learning approach helps to remove the variation from machine learning algorithm selection and the differences in expertise between groups.

In recent years, deep learning-based radiomics have been rapidly developed. Whereas other techniques are generally based on predefined features, deep learning methods allow for automatic extraction of high-level features by using multiple layers of neural networks that imitate the functions of the human visual network.^3,48^ While this technique has the advantage of not requiring image segmentation or predefined features, it also increases the difficulty in performing step-wise quality control due to the intrinsic nature of complex, high-integrated neural network layers. In addition, deep learning techniques often need significantly larger datasets compared to traditional machine learning techniques to reduce the chance of over-fitting due to a much greater number of features generated within the neural networks. The technique of transfer learning can help reduce the data requirement by using an artificial neural network model generated for another similar task as a basis for a new task.^49^

### Radiomic Model Validation

Since many commonly used machine learning algorithms can train a large number of features in proportion to the size of data, it is important to minimize the risk of over-fitting. At the minimum, the trained models should be evaluated in the internal dataset by cross-validation methods. Ideally, further validation using independent external cohorts can further strengthen the credibility of the radiomic models.^50,51^ In order for this validation to be meaningful, it is necessary to ensure that the study populations of the internal and external datasets are comparable. The details of both populations should be clearly described. Similarly, the reference standard used to supervise the training of a machine learning model should also be comparable to that used in the validation population.

## Pros and Cons of Standardization

There are several benefits to the standardization of radiomic methodology. When imaging acquisition, preprocessing, and postprocessing steps are standardized, the variations of calculated radiomic features will be reduced, and the performance of radiomic models will be improved. Besides improving interoperability and reproducibility, standardization can help define the processes of pooling results from multiple institutions and reduce the chances of incompatible datasets that would lower the accuracy of radiomic models. By defining the methodology and reporting guidelines, a growing body of literature with standardized nomenclature can facilitate comparison of results.^52^

However, is standardization always good? The fields of AI and radiomics are rapidly developing and consistently changing. New emerging techniques and algorithms are being published and implemented rapidly. By forcing researchers and organizations to follow a strict guideline, this may only be effective if the guidelines are updated to reflect the current development. If the guidelines are outdated, such efforts for standardization can potentially hinder innovation. Since technology is constantly improving, today’s “standard” is likely going to be different from tomorrow. For these concerns, collaborative efforts should be dedicated to constantly reviewing and updating standard protocols.

Another potential downside for standardization is a more narrow range of imaging parameters or postprocessing methods that can limit the discovery of clinically useful biomarkers from “nonstandard” protocols. In other words, standardization facilitates the validation side of efforts but may impede the exploration of novel features. If the size of data collection is sufficiently large, a likely scenario due to ongoing efforts in open data sharing, this may obviate the need for restricting imaging parameters and allow a certain degree of data heterogeneity.

## Current Efforts to Improve Standardization

Standardization efforts are being investigated throughout the field of AI. Leading organizations such as ISO and IEEE are involved in a broader sense as governing bodies.^53^ Within neuroimaging, the American Society of Neuroradiology has initiated a task force to develop training and educational programs related to AI in neuroimaging.^54^ For example, one branch is addressing quality assurance issues in regard to standardization, reliability, and reproducibility and also working in conjunction with the National Institute of Standards and Technology.

Within the scope of cancer biomarker development, the most comprehensive effort to standardize the extraction of image biomarkers comes from Image Biomarker Standardization Initiative (IBSI). The IBSI helps define some of the reporting guidelines.^52^ The main challenge according to these initiatives is to help solidify the process of translating acquired imaging into high-throughput image markers. Before this initiative, there is felt to be a lack of available consensus-based guidelines. This effort provides guidelines into standardized image biomarker nomenclature and definitions, general image processing workflow, tools for software implementation, and reporting guidelines. Another article by the Cancer Research UK and the European Organization for Research and Treatment of Cancer described the creation of an imaging biomarker roadmap for cancer studies by assembling a group of experts to discuss the challenges of imaging biomarker validation and standardization.^55^

Due to existing variations in imaging hardware, standardization of imaging acquisition is often challenging. Most ongoing efforts are to minimize acquisition variations by defining a standard range of acquisition parameters for major equipment vendors or platforms. For example, brain tumor imaging protocol (BTIP) was developed as a standard brain MRI protocol for imaging of primary brain gliomas^56^ that are increasingly adopted in recent clinical trials. This standard protocol includes a 3D T1-weighted sequence that was adopted from a preexisting standard imaging sequence used for imaging of Alzheimer’s disease.^57^ More recently, BTIP-BM has been proposed as standard protocol for assessment of brain metastases.^58^ Similar imaging acquisition criteria are created for PET imaging for glioma.^59^ Here the challenge is in making a standard protocol that clinical groups of different practice settings are willing to adopt without clear proven clinical benefit. Thus, if a new standard protocol can be modified from another existing, commonly used standard protocol it will allow easier implementation.

The IBSI also helps to make sure that the feature values from different groups follow the same feature definition. There are open-source tools that confirm to the IBSI such as pyradiomics.^60^ Calculators, such as RadCaT, help provide a calculation for a large number of radiomic features that are in compliance with IBSI standards.^7^ Imaging biomarker explorer is a software platform that is designed to implement common radiomic tasks.^61^ This software has also been shown to be highly compliant to the IBSI standards.^62^ LIFEx is a radiomic feature calculator designed for multiple modalities including PET, SPECT, MRI, CT, and ultrasound which is at least partly compliant with IBSI standards.^63^ Brain Cancer Imaging Phenomics Toolkit (brain-CaPTk) is developed as a type of cancer imaging phenomics toolkit for quantitative imaging analytics for precision diagnostics and predictive modeling of clinical outcome.^64,65^ Even with the development of these software tools, there may still be significant variations in features among software packages and indicates the need for standardization.^66^ Recently, the IBSI standardized a set of 169 radiomic features, which could verify and calibrate different radiomics software.^67^

The creation of open-access high-quality imaging datasets has been promoted as a way to improve the model training and validation process.^55^ A popular repository that encourages the sharing of data is The Cancer Imaging Archive.^68^ One paper recommends at least 10–15 patients for each feature evaluated.^69^ Having sufficient high-quality public data can make this task much easier.

Despite the best effort for researchers to adopt these strategies to reduce variability, it is often not possible to completely control the numerous variables within each step in developing a machine learning-based imaging biomarker. Investigators should still provide details of all essential steps from acquisition to pre- and postprocessing in accordance with a standard reporting guideline (ie, IBSI) to maximize the ability for other researchers to reproduce research results.

## Conclusions

Today, despite rapid advances in the development of AI technologies, major research organizations that perform AI research are often absent from the efforts in the standardization of imaging biomarker methodology.^53^ Whether the research is performed in the industry or within academic institutions, successful AI and radiomics applications in clinical medicine require a clear demonstration of their reproducibility and generalizability. With greater collaborative efforts among different institutions for collecting data such as from oncology patients, standardization of data acquisition and analysis methods may facilitate research results to be interoperable and reliable for integration into different practice environments.

## Funding

None.



**Conflict of interest statement.** None.



**Authorship Statement.** Writing and revision: X.T.L. and R.Y.H.
